# Bayesian latent class analysis produced diagnostic accuracy estimates that were more interpretable than composite reference standards for extrapulmonary tuberculosis tests

**DOI:** 10.1186/s41512-022-00125-x

**Published:** 2022-06-16

**Authors:** Emily L. MacLean, Mikashmi Kohli, Lisa Köppel, Ian Schiller, Surendra K. Sharma, Madhukar Pai, Claudia M. Denkinger, Nandini Dendukuri

**Affiliations:** 1 McGill International TB Centre, Research Institute of the McGill University Health Centre, Montréal, Canada; 2 Department of Epidemiology, Biostatistics and Occupational Health, McGill University, Montréal, Canada; 3 FIND, Geneva, Switzerland; 4 Division of Tropical Medicine, Center of Infectious Diseases, Heidelberg University, Heidelberg, Germany; 5 Department of Medicine, McGill University Health Centre, Montréal, Canada; 6 Department of Medicine, All India Institute of Medical Sciences, New Delhi, India

**Keywords:** Tuberculosis meningitis, Tuberculosis lymphadenitis, Tuberculosis pleuritis, Bayes theorem, Sensitivity and specificity

## Abstract

**Background:**

Evaluating the accuracy of extrapulmonary tuberculosis (TB) tests is challenging due to lack of a gold standard. Latent class analysis (LCA), a statistical modeling approach, can adjust for reference tests’ imperfect accuracies to produce less biased test accuracy estimates than those produced by commonly used methods like composite reference standards (CRSs). Our objective is to illustrate how Bayesian LCA can address the problem of an unavailable gold standard and demonstrate how it compares to using CRSs for extrapulmonary TB tests.

**Methods:**

We re-analyzed a dataset of presumptive extrapulmonary TB cases in New Delhi, India, for three forms of extrapulmonary TB. Results were available for culture, smear microscopy, Xpert MTB/RIF, and a non-microbiological test, cytopathology/histopathology, or adenosine deaminase (ADA). A diagram was used to define assumed relationships between observed tests and underlying latent variables in the Bayesian LCA with input from an inter-disciplinary team. We compared the results to estimates obtained from a sequence of CRSs defined by increasing numbers of positive reference tests necessary for positive disease status.

**Results:**

Data were available from 298, 388, and 230 individuals with presumptive TB lymphadenitis, meningitis, and pleuritis, respectively. Using Bayesian LCA, estimates were obtained for accuracy of all tests and for extrapulmonary TB prevalence. Xpert sensitivity neared that of culture for TB lymphadenitis and meningitis but was lower for TB pleuritis, and specificities of all microbiological tests approached 100%. Non-microbiological tests’ sensitivities were high, but specificities were only moderate, preventing disease rule-in. CRSs’ only provided estimates of Xpert and these varied widely per CRS definition. Accuracy of the CRSs also varied by definition, and no CRS was 100% accurate.

**Conclusion:**

Unlike CRSs, Bayesian LCA takes into account known information about test performance resulting in accuracy estimates that are easier to interpret. LCA should receive greater consideration for evaluating extrapulmonary TB diagnostic tests.

**Supplementary Information:**

The online version contains supplementary material available at 10.1186/s41512-022-00125-x.

## Background

Extrapulmonary tuberculosis (TB) comprised approximately 16% of the global TB burden in 2019, or 1.6 million cases [1]. This estimate is highly uncertain as a reliable “gold standard” to diagnose extrapulmonary TB cases is unavailable. The requisite non-respiratory samples are difficult to obtain, and existing diagnostic tests are not optimized for these typically paucibacillary samples. Mycobacterial culture and sputum smear microscopy, the conventional microbiological tests for TB, cannot detect bacteria at low counts, although cultures are substantially more sensitive than smears. Similarly, the limit of detection of Xpert MTB/RIF (Xpert) (Cepheid, USA), the World Health Organization-endorsed molecular test, is too high to detect TB in samples with low numbers of bacteria [2]. Consequently, multiple microbiological and clinical tests often requiring invasive techniques are relied on to make a diagnosis, with each test’s accuracy varying by extrapulmonary specimen type [3]. Therefore, when evaluating the performance of a new extrapulmonary TB test, the conventional tests cannot be treated as perfect reference standards as this will lead to bias [4].

In response, a composite reference standard (CRS) that combines results from multiple tests and clinical assessments in some pre-defined way is often employed to classify individuals as extrapulmonary TB-positive or –negative [5–8]. Though CRSs are designed with the goal of improving upon the accuracy of the individual component tests, it is recognized that they are imperfect themselves and, moreover, they have been criticized for making sub-optimal use of gathered data [9, 10]. Specifically, the most commonly used CRSs ignore the sensitivity and specificity of individual assessment components (i.e., tests and clinical symptoms) and treat them all as having similar accuracy. CRSs also assume that the component tests are independent of each other. However, it is possible that different imperfect tests are conditionally dependent, meaning that multiple tests may be more likely to be simultaneously falsely negative or falsely positive than if they were independent. For example, given all microbiological tests for extrapulmonary TB rely on bacterial load, they may all produce false negative results for a paucibacillary extrapulmonary TB-positive individual [10].

Latent class analysis (LCA) is a statistical modeling solution to address these issues [11]. LCA can model the accuracy of each imperfect diagnostic test, as well as dependence between tests, to simultaneously estimate disease prevalence and the sensitivity and specificity of all tests at hand [10, 12]. Bayesian LCA can further include reliable prior information on disease prevalence or test accuracy parameters, when available, e.g., high specificity values for microbiological tests. It has been applied to estimate diagnostic test accuracy for latent TB infection [13], childhood pulmonary TB [14], chlamydia [15], and *Helicobacter pylori* infection [16], among other diseases.

Extrapulmonary TB has traditionally received less attention than pulmonary TB owing to its less infectious nature. Nonetheless, it presents a significant burden to healthcare systems and patients, particularly due to the difficulty in diagnosis, and the risk of severe outcomes with certain extrapulmonary TB forms is high (e.g., TB meningitis and miliary TB). LCA may overcome limitations of naïve methods to produce better prevalence and test accuracy estimates, better approximating the true burden. However, it must be acknowledged that LCA methods are considered methodologically complex and difficult to validate [11].

Therefore, to illustrate the steps involved in conducting an LCA and compare with CRS, we re-analyzed an existing dataset of extrapulmonary samples from adults with presumptive extrapulmonary TB [17]. The original study evaluated Xpert accuracy using a series of CRSs for TB meningitis, TB lymphadenitis, and TB pleuritis. Resultant estimates of Xpert sensitivity, specificity, and disease prevalence varied widely depending on the CRS used [17], making them difficult to interpret. Our objective was to use Bayesian LCA to estimate the diagnostic accuracy of all the available tests and to discuss the advantages and challenges of this approach.

## Methods

### Primary dataset details

The primary dataset comprised all extrapulmonary samples from adults with presumptive extrapulmonary TB received by the All-India Institute of Medical Sciences (AIIMS), a tertiary hospital in New Delhi, India in 2012 [17]. No participants had taken anti-TB therapy (ATT) for longer than 2 weeks. All samples underwent testing with Xpert, an automated PCR-based assay that detects mycobacterial-specific DNA; BACTEC Mycobacteria Growth Indicator Tube (Becton Dickinson, USA) liquid culture or Lowenstein-Jensen solid culture; and Ziehl-Neelsen (acid-fast bacilli) sputum smear microscopy. As conventional pulmonary TB tests perform sub-optimally for extrapulmonary TB, non-specific assays are also deployed in conjunction with TB testing [18]. Regarding non-microbiological tests, for presumptive TB lymphadenitis, results were available from cytopathology/histopathology, wherein local cells or tissues are examined for pathological patterns such as caseating necrosis [19], while for presumptive TB meningitis and pleuritis (solid tissue or fluid), levels of deaminase (ADA), an enzyme expressed in leukocytes associated with granulatomous reactions [20], were available. Additionally, for each participant, type of extrapulmonary sample tested (indicating extrapulmonary TB form), resistance to rifampicin, initiation of ATT, and treatment response were available. Each participant had one result per test. Demographic covariates and clinical symptoms were unavailable. As this was a secondary analysis of previously collected data which had received ethical approvals and informed consent from all participants, ethical approval was not necessary.

In the original publication [17], the authors focused on the then-novel Xpert test, reporting its sensitivity and specificity against culture. Recognizing culture’s imperfect performance, they subsequently compared Xpert to a series of CRSs, resulting in multiple estimates of Xpert sensitivity and specificity.

### Latent class model specification

#### Diagrammatic representation

**Fig. 1:**
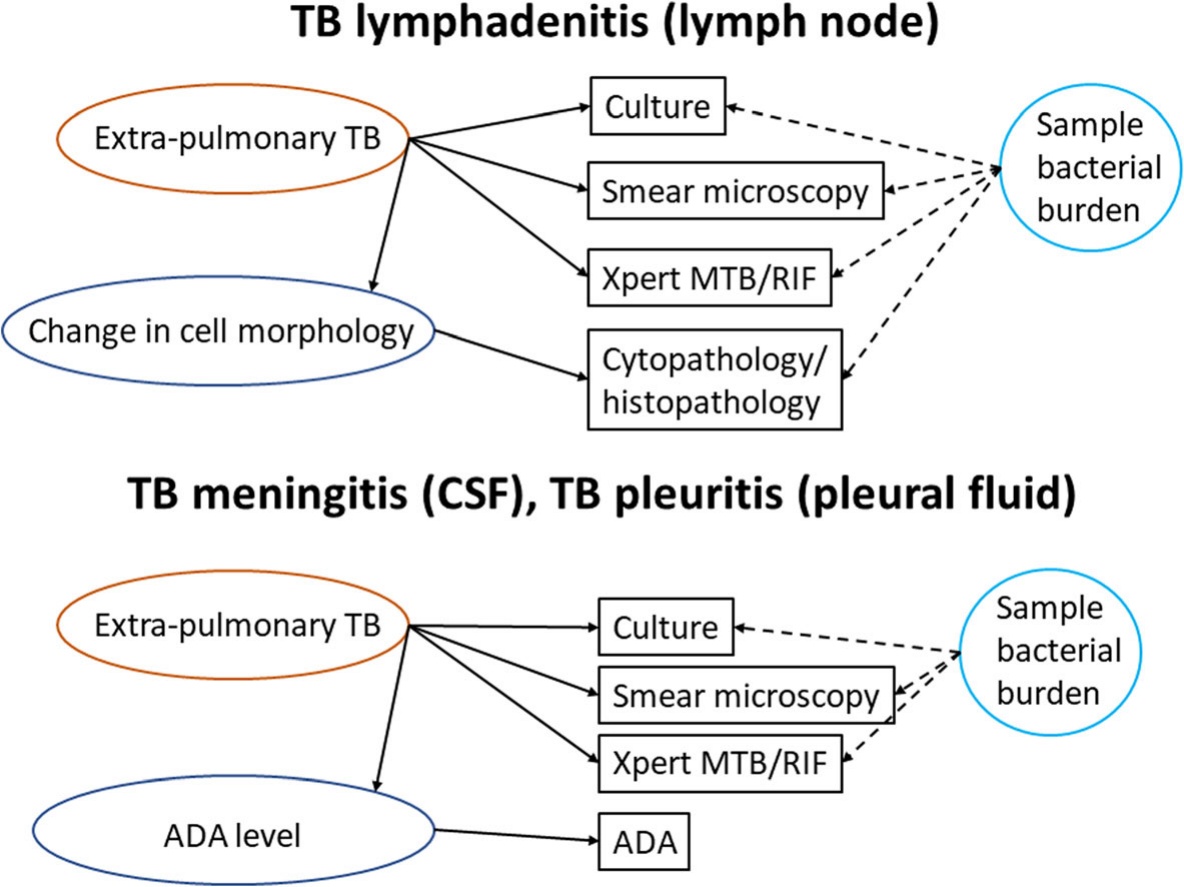
Heuristic model. The model shows the assumed relationships between latent classes (ovals), diagnostic test results (rectangles), and random effect representing sample bacterial burden (circle). *ADA* adenosine deaminase, *CSF* cerebral spin fluid, *TB* tuberculosis

We first created heuristic diagrams for each extrapulmonary TB form (meningitis, lymphadenitis, pleuritis) to illustrate our assumptions about the relationships between observed test results and unobservable (latent) extrapulmonary TB status [14] (Fig. 1). These diagrams identify the measurand of each test, i.e., the quantity it measures. Culture, smear, and Xpert use different techniques to measure the presence of *Mycobacterium tuberculosis* in the extrapulmonary sample. We assumed that this was equivalent to their measurand being extrapulmonary TB itself. In the case of TB meningitis and pleuritis, the ADA test was also deployed. The ADA test result is determined by the latent variable “ADA level” and not by extrapulmonary TB. Similarly, in the situation of TB lymphadenitis, the result on cytopathology/histopathology is determined by “change in cell morphology” rather than extrapulmonary TB. The nonspecific tests, ADA test and cytopathology/histopathology, do not measure the target condition extrapulmonary TB per se; instead, their measurands are signals (e.g., inflammation) caused by extrapulmonary TB or other diseases. Therefore, for each of the LCA models, we assume that there are four possible latent classes resulting from combinations of the latent measurands: (1) Extrapulmonary TB-positive, non-specific measurand-positive; (2) extrapulmonary TB-positive, non-specific measurand-negative; (3) extrapulmonary TB-negative, non-specific measurand-positive; and (4) extrapulmonary TB-negative, non-specific measurand-negative. This approach differs from the widely used two-class LCA model which assumes all observed tests measure the target condition, i.e., extrapulmonary TB in our applications. We preferred the four-class LCA as it leads to greater interpretability of the latent classes. The parameters resulting from a two-class LCA (prevalence of extrapulmonary TB and accuracy of tests with respect to extrapulmonary TB) may be obtained easily as a subset of the four-class LCA.

Observation with smear microscopy or cytopathology/histopathology, bacterial growth on culture, and detection of mycobacterial DNA using Xpert are all increasingly likely as bacterial burden increases. Contrastingly, in people with paucibacillary extrapulmonary TB, these tests will tend to show negative results. We thus hypothesized that the underlying bacterial burden may create conditional dependence between test results, i.e., dependence between tests in the first two latent classes of extrapulmonary TB positive subjects [21], even though tests are based on different mechanisms (Fig. 1).

#### Statistical model

The observed diagnostic test results were assumed to be a mixture of results from the four underlying latent classes. The unknown parameters of the model were the prevalence of the four latent classes and each test’s sensitivity and specificity with respect to its measurand. Using these parameters, we can further determine the prevalence of each measurand, the accuracy of the non-specific measurand, and the accuracy of the non-specific test for extrapulmonary TB. For example, the prevalence of extrapulmonary TB can be obtained by adding the prevalence of the two classes with extrapulmonary TB, with or without the non-specific condition. We introduced a random effect, corresponding to sample bacterial burden, to account for the conditional dependence among microbiological tests and cytopathology/histopathology in the group of people with extrapulmonary TB. In people without extrapulmonary TB, all test outcomes were considered conditionally independent. Individuals with invalid or missing test results were assumed to be missing at random and retained in the analysis, with missing test results imputed by Bayesian imputation.

### Bayesian model estimation

Using a four-class rather than a two-class approach increases the number of unknown parameters in the model and increases concerns for non-identifiability, i.e., the lack of a unique solution to the model. By constraining each test’s accuracy parameters to be determined only by its measurand, we limited the number of parameters added, resulting in fewer parameters to be estimated than available degrees of freedom for all three forms of extrapulmonary TB (see Supplemental methods for details on identifiability).

We used a Bayesian approach to fit the latent class models (see Supplement for model likelihoods and prior distributions). As the posterior distributions of the parameters of interest (sensitivity, specificity, prevalence) could not be computed analytically, we sampled from the posterior distributions using a Markov Chain Monte Carlo (MCMC) approach with the rjags package (Version 4-8) through Rstudio (Version 3.5.2). Non-informative priors were used for all models with truncated prior distributions for the non-specific tests’ sensitivities and specificities to contain them above 50% and avoid label switching (mirror solutions) (see Supplement). The Supplement contains details of model specifications and sampling, and further programs for data preparation and model checking are available in a repository: https://osf.io/9wdb3/?view_only=730fb3e7d9114405bb51075748703054.

### LCA model validation

There is no ideal way to validate the results of an LCA due to the lack of a perfect reference test. As in previous work [14, 15], we used an indirect approach. For each test pattern, we compared the observed frequency of receiving ATT with the LCA-derived probability of disease. If the LCA was valid, we expected to observe that as the LCA-derived probability of TB increased, the probability of ATT would also increase.

### Composite reference standards

We used the same definitions for the series of CRSs as the original publication [17]. “CRS 1+” was defined as any one component test of the CRS being positive versus all four tests being negative; “CRS2+” was defined as any two component tests of the CRS being positive versus all four tests being negative; and so on to CRS 4+. For each extrapulmonary TB form, the latent class model was used to estimate the sensitivity and specificity of each CRS. Each individual’s probability of disease, as computed by LCA, was used as the reference standard. The Supplement contains the expressions and code used for these computations.

## Results

### Dataset description

The original study had 1376 samples. After excluding specimens for five forms of extrapulmonary TB not considered in the current analyses, there remained 299 lymph node samples for TB lymphadenitis, 388 pleural fluid samples for TB pleuritis, and 230 cerebral spinal fluid (CSF) samples for TB meningitis.

### Bayesian latent class analysis: estimated prevalence and diagnostic accuracies

Estimates from the latent class analysis (median values and 95% credible intervals (CrI)) of sensitivity and specificity for each diagnostic test are shown in Table 1, organized by form of extrapulmonary TB. Prevalence of TB lymphadenitis, TB meningitis, and TB pleuritis were estimated as 60.5% (95%CrI: 54.1–67.9), 15.9% (95%CrI: 9.70–24.3), and 35.3% (95%CrI: 26.7–48.8), respectively. Note that these are not population-level estimates, but rather prevalence estimates among recruited participants at a tertiary care facility. Supplementary Table S1 shows the probabilities of each of the four latent classes.

**Table 1:** Bayesian latent class analysis-derived diagnostic accuracies of tests for each form of extrapulmonary TB

	Culture	Xpert	Smear microscopy	Cytopathology/Histopathology	ADA
TB lymphadenitis					
Sensitivity (%) (95% CrI)	90.1 (80.3, 95.4)	86.6 (77.2, 92.2)	26.8 (20.6, 33.7)	98.7 (96.1, 99.7)	NA
Specificity (%) (95% CrI)	99.3 (96.2,100)	98.5 (94.4, 100)	99.4 (97.0, 100)	83.5 (74.6, 93.2)	NA
TB meningitis					
Sensitivity (%) (95% CrI)	60.5 (43.2, 82.7)	52.6 (36.2, 73.3)	27.5 (14.9, 42.6)	NA	83.1 (64.8, 94.5)
Specificity (%) (95% CrI)	99.2 (96.8, 100)	99.5 (97.6, 100)	98.6 (96.3, 99.7)	NA	90.7 (83.8, 98.1)
TB pleuritis					
Sensitivity (%) (95% CrI)	75.4 (56.1, 94.5)	37.7 (27.2, 49.8)	15.4 (9.4, 23.4)	NA	94.6 (88.8, 98.1)
Specificity (%) (95% CrI)	99.4 (97.3, 100)	96.9 (93.8, 99.0)	99.3 (97.7, 99.9)	NA	74.7 (65.1, 90.3)

Lymph node samples were used for TB lymphadenitis; CSF samples were used for TB meningitis; pleural fluid samples were used for TB pleuritis. The performance of all tests in this table is the estimates with respect to the target condition, extrapulmonary TB

*ADA* adenosine deaminase, *CrI* credible interval, *CSF* cerebral spinal fluid, *NA* not applicable, *TB* tuberculosis

Test performance varied by specimen type, as previously observed [3, 22–24]. Culture and Xpert sensitivities were highest for TB lymphadenitis, and lower for TB meningitis and TB pleuritis. Sensitivity of ADA and cytopathology/histopathology with respect to extrapulmonary TB were high, but imperfect specificities prevented disease rule-in. As expected, sensitivity and specificity point estimates of these tests were higher with respect to their measurands than with respect to the target condition, extrapulmonary TB (Table 2). Specificities for culture, Xpert, and smear were universally almost perfect.

**Table 2:** Cytopathology/histopathology and ADA test performance with respect to each extrapulmonary TB form and measurands

	Cytopathology/histopathology with respect to EPTB form	ADA test with respect to EPTB form	Cytopathology/histopathology with respect to measurand	ADA test with respect to measurand
TB lymphadenitis				
Sensitivity (%) (95% CrI)	98.7 (96.1, 99.7)	NA	99.8 (98.2, 100)	NA
Specificity (%) (95% CrI)	83.5 (74.6, 93.2)	NA	91.7 (79.6, 99.5)	NA
TB meningitis				
Sensitivity (%) (95% CrI)	NA	83.1 (64.8, 94.5)	NA	91.9 (73.3, 99.6)
Specificity (%) (95% CrI)	NA	90.7 (83.8, 98.1)	NA	95.0 (86.5, 99.8)
TB pleuritis				
Sensitivity (%) (95% CrI)	NA	94.6 (88.8, 98.1)	NA	97.5 (91.9, 98.1)
Specificity (%) (95% CrI)	NA	74.7 (65.1, 90.3)	NA	86.8 (70.1, 99.4)

The performance of each test with respect to type of EPTB, otherwise referred to as the target condition, and with respect to the measurand is provided. Regarding non-specific tests ADA and cytopathology/histopathology, we have discerned between their performance at measuring their measurands versus the target condition, EPTB. This parametrization more accurately captures the nuance of the testing scenario, as ‘target condition’ and ‘measurand’ are not interchangeable entities [25]. Reassuringly, they both performed better at measuring their measurand (ADA level or change in cell morphology) than measuring EPTB

*ADA* adenosine deaminase, *CrI* credible interval, *EPTB* extrapulmonary TB, *NA* not applicable, *TB* tuberculosis

### Composite reference standard analysis

As in the earlier publication [17], we confirmed that the four composite reference standards provided four estimates of Xpert accuracy for each form extrapulmonary TB. Regardless of the extrapulmonary TB form, when disease-positivity was defined by the presence of any one positive test result, CRS 1+ classified most individuals as disease positive and therefore had the highest sensitivity and lowest specificity (Table 3). This was observed across extrapulmonary TB forms. Correspondingly, with increasingly stringent CRS definition of disease-positivity, the sensitivities declined across all disease forms, while specificities increased, as the number of false positives decreased.

**Table 3:** Diagnostic accuracy of a series of composite reference standards for each form of extrapulmonary TB

	CRS 1+	CRS 2+	CRS 3+	CRS4+
		TB lymphadenitis		
Sensitivity (%) (95% CI)	99.8 (97.0, 100)	97.1 (89.1, 99.5)	81.8 (74.8, 84.2)	26.4 (24.2, 27.2)
Specificity (%) (95% CI)	83.9 (80.9, 92.3)	99.6 (98.4, 100)	100 (100, 100)	100 (100, 100)
		**TB meningitis**		
Sensitivity (%) (95% CI)	95.0 (84.6, 99.6)	64.4 (46.3, 87.1)	44.4 (31.3, 61.9)	17.8 (12.5, 24.8)
Specificity (%) (95% CI)	88.3 (84.8, 94.1)	98.8 (97.8, 99.9)	100 (100, 100)	100 (100, 100)
		**TB pleuritis**		
Sensitivity (%) (95% CI)	98.9 (96.0, 99.9)	75.5 (56.3, 94.8)	31.1 (23.1, 39.2)	9.00 (6.70, 11.4)
Specificity (%) (95% CI)	73.7 (67.2, 88.0)	99.3 (97.9, 100)	100 (99.8, 100)	100 (100, 100)

CRS 1+ indicates any one positive test in CRS versus all four parameters being negative. CRS 2+: any two tests positive in CRS versus all four parameters being negative. CRS 3+: any three tests positive in CRS versus all four parameters being negative. CRS 4+: all four tests positive in CRS versus all four parameters being negative [17]. Lymph node samples were used for TB lymphadenitis; CSF samples were used for TB meningitis; pleural fluid samples were used for TB pleuritis

*CI* confidence interval, *CRS* composite reference standard, *CSF* cerebral spinal fluid, *TB* tuberculosis

### Model fit

As shown in Tables 4, 5, and 6, model-derived counts for all observed test result patterns generally resembled observed frequencies, indicating that the data did not deviate from the proposed model. There was no evidence that correlation residuals deviated significantly from 0, implying there was no evidence of unaccounted conditional dependence; see Supplemental results, Fig. S1, and Fig. S2 for further details on model fit.

**Table 4:** Observed counts, expected counts, and TB lymphadenitis probability by test result pattern

Culture	Xpert	Smear	Cytopathology/histopathology	No. observed	No. expected by LCA (95% CrI)	Probability of TB lymphadenitis by LCA (95% CrI)	Probability of TB lymphadenitis by CRS			
							CRS1+	CRS2+	CRS3+	CRS4+
−	−	−	−	96	92 (85, 98)	0.003 (0, 0.08)	0	0	0	0
−	−	−	+	21	22 (15, 29)	0.18 (0.04, 0.73)	1	0	0	0
−	+	−	−	1	2 (0, 6)	0.18 (0.01, 0.89)	1	0	0	0
−	+	−	+	10	9 (5, 15)	0.98 (0.85, 0.99)	1	1	0	0
+	−	−	+	15	14 (8, 20)	0.99 (0.94, 0.99)	1	1	0	0
+	−	+	+	2	2 (1, 5)	1.00 (0.99, 1)	1	1	1	0
+	+	−	+	85	86 (75, 98)	1.00 (0.99, 1)	1	1	1	0
+	+	+	−	1	0 (0, 1)	1.00 (0.99, 1)	1	1	1	0
+	+	+	+	42	40 (31, 49)	1.00 (0.99, 1)	1	1	1	1
NA	−	−	−	1	NE	0.002 (0, 0.01)	NE	0	0	0
NA	−	−	+	1	NE	0.47 (0.28, 0.85)	1	NE	0	0
NA	+	−	+	13	NE	1 (0.99, 1)	1	1	NE	0
NA	+	+	+	4	NE	1 (0.99, 1)	1	1	1	NE
NA	NA	−	NA	7	NE	0.53 (0.45, 0.61)	NE	NE	NE	0

Tests were all performed in lymph node samples (*n* = 299). A “+” indicates positive test result and “−” indicates negative test result. CRS 1+ indicates any one positive test in CRS versus all four parameters being negative. CRS 2+: any two tests positive in CRS versus all four parameters being negative. CRS 3+: any three tests positive in CRS versus all four parameters being negative. CRS 4+: all four tests positive in CRS versus all four parameters being negative

*CrI* credible interval, *LCA* latent class analysis, *NA* value not available, *NE* result not estimable, *No*. number of, *Smear* smear microscopy, *TB* tuberculosis

**Table 5:** Observed counts, expected counts, and TB meningitis probability by test result pattern

Culture	Xpert	Smear	ADA	No. observed	No. expected by LCA (95% CrI)	Probability of TB meningitis by LCA (95% CrI)	Probability of TB meningitis by CRS			
							CRS1+	CRS2+	CRS3+	CRS4+
−	−	−	−	170	166 (158,173)	0.01 (0, 0.04)	0	0	0	0
−	−	−	+	27	29 (22,37)	0.33 (0.08, 0.76)	1	0	0	0
−	−	+	−	2	2 (1,6)	0.004 (0, 0.07)	1	0	0	0
−	+	−	+	3	2 (1,4)	0.96 (0.06, 0.99)	1	1	0	0
+	−	−	−	1	2 (1,6)	0.30 (0.03, 0.93)	1	0	0	0
+	−	−	+	6	4 (2,7)	0.97 (0.08, 1)	1	1	0	0
+	+	−	+	7	6 (4,10)	1.0 (0.99, 1)	1	1	1	0
+	+	+	−	2	1 (0,3)	1.0 (0.99, 1)	1	1	1	0
+	+	+	+	6	6 (4,9)	1.0 (0.99, 1)	1	1	1	1
−	−	−	NA	6	NE	0.03 (0, 0.08)	NE	0	0	0

Tests were all performed in CSF samples (*n* = 230). A “+” indicates positive test result and “−” indicates negative test result. CRS 1+ indicates any one positive test in CRS versus all four parameters being negative. CRS 2+: any two tests positive in CRS versus all four parameters being negative. CRS 3+: any three tests positive in CRS versus all four parameters being negative. CRS 4+: all four tests positive in CRS versus all four parameters being negative

*ADA* adenosine deaminase, *CrI* credible interval, *CSF* cerebral spinal fluid, *LCA* latent class analysis, *NA* value not available, *NE* result not estimable, *No*. number of, *Smear* smear microscopy, *TB* tuberculosis

**Table 6:** Observed counts, expected counts, and TB pleuritis probability by test result pattern

Culture	Xpert	Smear	ADA	No. observed	No. expected by LCA (95% CrI)	Probability of TB pleuritis by LCA (95% CrI)	Probability of TB pleuritis by CRS			
							CRS1+	CRS2+	CRS3+	CRS4+
−	−	−	−	180	175 (162, 185)	0.008 (0, 0.03)	0	0	0	0
−	−	−	+	84	86 (75, 98)	0.33 (0.06, 0.78)	1	0	0	0
−	−	+	−	1	1 (0, 4)	0.005 (0, 0.07)	1	0	0	0
−	+	−	−	4	6 (2, 11)	0.016 (0, 0.10)	1	0	0	0
−	+	−	+	4	4 (2, 7)	0.54 (0.05, 0.92)	1	1	0	0
+	−	−	−	1	4 (1, 7)	0.70 (0.2, 0.99)	1	0	0	0
+	−	−	+	50	45 (36, 53)	0.99 (0.96, 1)	1	1	0	0
+	−	+	+	3	3 (1, 6)	1.0 (0.99, 1)	1	1	1	0
+	+	−	−	2	1 (0, 3)	0.98 (0.81, 0.99)	1	1	0	0
+	+	−	+	24	26 (20, 33)	1.0 (0.99, 1)	1	1	1	0
+	+	+	+	11	12 (8, 16)	1.0 (0.99, 1)	1	1	1	1
NA	−	−	−	3	NE	0.02 (0.01, 0.05)	NE	0	1	0
NA	−	−	+	7	NE	0.53 (0.33, 0.84)	1	NE	0	0
NA	+	−	−	1	NE	0.29 (0.10, 0.62)	1	NE	0	0
NA	+	−	+	6	NE	0.98 (0.96, 1)	1	1	NE	0
NA	+	+	−	1	NE	0.59 (0.28, 0.93)	1	1	NE	0
NA	+	+	+	4	NE	0.99 (0.98, 1)	1	1	1	NE
NA	+	+	NA	1	NE	0.99 (0.96, 1)	1	1	NE	NE
NA	NA	−	NA	1	NE	0.32 (0.23, 0.45)	NE	NE	NE	0

Tests were all performed in pleural fluid samples (*n* = 388). A “+” indicates positive test result and “−” indicates negative test result. CRS 1+ indicates any one positive test in CRS versus all four parameters being negative. CRS 2+: any two tests positive in CRS versus all four parameters being negative. CRS 3+: any three tests positive in CRS versus all four parameters being negative. CRS 4+: all four tests positive in CRS versus all four parameters being negative

*ADA* adenosine deaminase, *CrI* credible interval, *LCA* latent class analysis, *NA* value not available, *NE* result not estimable. *No*. number of, *Smear* smear microscopy, *TB* tuberculosis

### Probability of extrapulmonary TB and association with probability of receiving ATT

Tables 4, 5 and 6 also display the LCA-derived probability of each extrapulmonary TB form for an individual with a particular test result pattern. In all three examples, many patterns were associated with high probability of extrapulmonary TB (close to 1) or a low probability (close to 0). The most difficult subjects to classify were those with a positive result only on the non-specific test. Contrastingly, using the CRSs, all subjects would be classified as diseased or non-diseased with 100% probability, regardless of the CRSs’ performance. Consider that for TB pleuritis (Table 6), individuals with positive results for Xpert and ADA but negative results for culture and smear would be classified as disease-positive by CRS1+ and CRS2+ but disease-negative by CRS3+ and CRS4+. Using LCA, their probability of having TB pleuritis was estimated as 0.54, reflecting the lack of certainty in their true classification based on the available evidence.

The observed probability of ATT was usually 100% whenever at least one test produced a positive result. This was true even when the calculated probability of extrapulmonary TB is low, an observation that was consistent across disease forms. For example, in CSF, with three microbiological tests negative but ADA positive, the probability of TB meningitis was only 33%, but the probability of ATT was 96% (26/27 patients). In pleural fluid, with all tests negative except Xpert, the probability of TB pleuritis was 1.6%, but all four patients with this test pattern received ATT. Thus, we did not find the probability of receiving ATT to be informative about the validity of the LCA models.

## Discussion

Producing correct estimates of diagnostic test accuracy is challenging without a perfect reference standard. We used Bayesian LCA to estimate multiple tests’ accuracies for three forms of extrapulmonary TB, along with disease prevalence. We also estimated the accuracy of a series of CRSs for these same conditions. By employing these two methods, we hope to demonstrate the utility of Bayesian LCA for evaluating diagnostic test accuracy. We observed that each test’s sensitivity varied by extrapulmonary TB form, and none was perfect. Specificities were generally very high with respect to extrapulmonary TB, except for ADA and cytopathology/histopathology. Culture sensitivity, often treated as 100%, was imperfect and only slightly higher than Xpert’s for TB lymphadenitis and meningitis; indeed, India’s Index-TB Guidelines recommend Xpert for these two disease forms [18]. Though there is no way to validate these models, the results were in-keeping with our expectations. For example, for all forms of extrapulmonary TB, we found that the sensitivity of culture was greater than the sensitivity of Xpert which, in turn, was greater than the sensitivity of smear, as would be expected based on the knowledge of the mechanisms behind these tests, while all of them had near perfect specificity. The non-specific tests always had higher accuracy with respect to the measurand they were designed to measure than with respect to extrapulmonary TB.

We calculated that no CRS was 100% accurate; rather, accuracy varied depending on the rule by which the CRS was defined. Further, there is no way of knowing which rule provided the true measure of disease status. With extrapulmonary TB, the diagnostic tests that comprise the CRSs are themselves imperfect, so assuming 100% sensitivity and specificity is unreasonable and ignores relevant biological information. Such use would result in biased index test accuracy estimates, with the true values obscure [10]. When using LCA, there is no assumption that reference tests perform perfectly. Instead, LCA incorporates all available tests results, concurrently adjusting for their unique accuracies and between-test dependence: this more comprehensive approach more closely approximates the real-world setting where each test brings a different quantum of information by the target condition. In doing so, LCA produces one figure that, based on stipulated assumptions, can be interpreted as the best estimate; for example, the latent class model estimated Xpert sensitivity for TB meningitis as 53% (95% CrI: 36–73). Contrast this with the original study, where the series of CRSs resulted in four different Xpert sensitivity estimates for TB meningitis, 33%, 50%, 70%, and 100% [17]. It is impossible for the reader to know which was the true measure of test accuracy. In this way, LCA-derived values are more clinically interpretable, as the reader does not need to discern between a series of values and select one as the best estimate.

In our study, we constructed a four-class latent class model as we felt it was more likely to achieve the desired separation into “extrapulmonary TB” and “not extrapulmonary TB.” Depending on the combination of observed test results, a two-class model may have resulted in the combining of two classes where either extrapulmonary TB or the non-specific measurand was present into one class, potentially leading to biased estimates of test accuracy [26]. We did fit the two-class latent model as well, for comparison (Tables S2 and S3). For TB lymphadenitis and TB meningitis, results from the two-class and four-class models were very similar because the two discordant classes had relatively low prevalence (Table S1). In the case of TB pleuritis, the two-class model gave a prevalence estimate that appeared equivalent to the probability of the latent classes where either extrapulmonary TB or ADA was positive (Table S2 and S3), resulting in slightly lower point estimates of the sensitivities of the microbiological tests and a slightly lower specificity of the ADA test.

We relied on a multi-disciplinary team of experts when creating our model, as goodness-of-fit metrics may fail to indicate model misspecification [27]. Consider that pathological signs on cytopathology/histopathology may be attributable to causes other than TB lymphadenitis. This means that individuals who had positive cytopathology/histopathology signals would be a mix of people with extrapulmonary TB and people with some other disease. Choosing a 4-class model allowed us to distinguish between conditions that could produce positive test results and prevented grouping a mix of extrapulmonary TB-positive and -negative people together in our “diseased” condition.

We found that the LCA-derived probability of extrapulmonary TB was not a good predictor for receiving ATT, as even in cases of low disease probability, patients received treatment. Seemingly, one, and certainly two, positive test results was sufficient to commence ATT. This is perhaps not surprising given the high TB prevalence in the study setting and the very high mortality risk of, for example, TB meningitis [28], so clinicians would rather “treat now and ask questions later.” When diagnosing extrapulmonary TB, clinicians also consider clinical variables that were unavailable in our dataset [18]. It is worth emphasizing that obtaining diagnostic test accuracy estimates is unlike making a clinical decision. Here, we have constructed a model to estimate test performance and have attempted to be transparent in unknowns, assumptions, and subjective choices, but other parameterizations are certainly possible.

### Strengths

We estimated the prevalence of three forms of extrapulmonary TB; understanding prevalence in a particular healthcare setting is critical to planning and policy making. Using LCA, we have made the best possible use of data by incorporating results from all available tests to determine sensitivities and specificities, while adjusting for the possibility of between-test dependence. Unlike with CRSs, we did not ignore any observed test results. Consider that for CRS1+, a single positive test result defines disease positivity; if there are three other negative test results, those three are functionally non-informative. Obtaining specimens for most forms of extrapulmonary TB is invasive and requires trained healthcare workers, so ensuring collected data are used to their best potential is an ethical decision.

### Limitations

First, as with any statistical model, the latent class models we have fit cannot be shown to be the true models. However, our models were reasonably well-specified, as evidenced by good agreement between observed and expected test result patterns and low residual correlation between test results. In some applications, external information, such as the proportion of patients with a given test pattern who were treated, provides useful information to validate the model. Here, this information was not very informative due to the sparse nature of the datasets. Second, LCA has been characterized as “black box-y” [29] and cautions have been raised that model misspecification is difficult to detect [27]. Certainly, its mechanisms are less intuitive to understand than Boolean decision rules like those often used when defining CRSs, but the theory underpinning LCA is well-defined and transparent [30, 31]. We have attempted to be clear by providing DAGs illustrating our assumptions of the relationships within the model. A final, general limitation is that parameter estimates depend on the available data. The available datasets had a small sample size and did not contain demographic or clinical assessment variables, resulting in poor precision of the estimates. Additionally, this prevented any relevant subgroup analyses.

## Conclusion

Basic methods like two-by-two table calculations and CRSs are known to produce imperfect estimates of diagnostic test accuracy. Latent class analysis, which can reflect knowledge of the individual tests used for diagnosis, should receive greater consideration in evaluating new tests’ performance.

## Supplementary Information

**Additional file 1: Supplemental Figure S1.** Pairwise residual correlation plots for tests run on A) lymph node, B) CSF, and C) pleural fluid. Test pair 1: culture and Xpert; 2: culture and smear microscopy; 3: culture and cytopathology/histopathology or ADA; 4: Xpert and smear microscopy; 5: Xpert and cytopathology/histopathology or ADA; 6: smear microscopy and cytopathology/histopathology or ADA. ADA – adenosine deaminase; CSF – cerebral spinal fluid; TB – tuberculosis. **Supplemental Figure S2.** Density plots. For each of the three models (one per form of extrapulmonary TB), density plots were generated for each parameter of interest. For test result parameters specificity, *C*, and sensitivity, *S*, the indices 1, …,4 indicate culture, Xpert, smear microscopy, and ADA or cytopathology/histopathology. **Supplementary Table S1.** Probabilities of the four latent classes, for each form of extrapulmonary TB as estimated by Bayesian latent class analysis. **Supplementary Table S2.** Diagnostic accuracies of tests for each form of extrapulmonary TB by latent class analysis. **Supplementary Table S3.** Prevalence of each form of extrapulmonary TB as estimated by the two-class latent class model

## Data Availability

The datasets analyzed during the current study are available in the Open Science Framework repository, https://osf.io/9wdb3/?view_only=730fb3e7d9114405bb51075748703054.

## Abbreviations

ADA: Adenoses deaminase
AIIMS: All India Institute of Medical Sciences
ATT: Anti-tuberculosis therapy
CI: Confidence interval
CrI: Credible interval
CRS: Composite reference standard
CSF: Cerebral spinal fluid
LCA: Latent class analysis
MCMC: Markov Chain Monte Carlo
NA: Not applicable
No.: Number of
Smear: Smear microscopy
TB: Tuberculosis
Xpert: Xpert MTB/RIF
