# Supplementary material for: Bayesian latent class analysis produced diagnostic accuracy estimates that were more interpretable than composite reference standards for extrapulmonary tuberculosis tests
Source: Diagn Progn Res. 2022 Jun 16;6:11. doi: 10.1186/s41512-022-00125-x (PMC9202094; doi:10.1186/s41512-022-00125-x)
Supplement: Supplementary file 1 — Additional file 1: Supplemental Figure S1. Pairwise residual correlation plots for tests run on A) lymph node, B) CSF, and C) pleural fluid. Test pair 1: culture and Xpert; 2: culture and smear microscopy; 3: culture and cytopathology/histopathology or ADA; 4: Xpert and smear microscopy; 5: Xpert and cytopathology/histopathology or ADA; 6: smear microscopy and cytopathology/histopathology or ADA. ADA – adenosine deaminase; CSF – cerebral spinal fluid; TB – tuberculosis. Supplemental Figure S2. Density plots. For each of the three models (one per form of extrapulmonary TB), density plots were generated for each parameter of interest. For test result parameters specificity, C, and sensitivity, S, the indices 1, …,4 indicate culture, Xpert, smear microscopy, and ADA or cytopathology/histopathology. Supplementary Table S1. Probabilities of the four latent classes, for each form of extrapulmonary TB as estimated by Bayesian latent class analysis. Supplementary Table S2. Diagnostic accuracies of tests for each form of extrapulmonary TB by latent class analysis. Supplementary Table S3. Prevalence of each form of extrapulmonary TB as estimated by the two-class latent class model [file 41512_2022_125_MOESM1_ESM.docx]

**SUPPLEMENT**

**Bayesian latent class analysis produced diagnostic accuracy estimates that were more interpretable than composite reference standards for extrapulmonary tuberculosis tests**

Emily L. MacLean^a,b^, Mikashmi Kohli^c^, Lisa Köppel^d^, Ian Schiller^e^, Surendra K. Sharma^f^, Madhukar Pai^a,b^, Claudia M. Denkinger^a,d^, Nandini Dendukuri^a,e,#^

**SUPPLEMENTAL METHODS**

***Latent class model: Likelihood and prior distributions***

Observed test results for the i^th^ subject (*i = 1,…, N*) on the j^th^ diagnostic test (*j=1,…,4*) are denoted by T_ij_. Test results take values 1 (for a positive test) and 0 (for a negative test).

Likelihood:

We assume:

T_ij_ ~ Bernoulli(p_ij_), where p_ij_ = $\sum_{Li=1}^{4} Pr(L_{i})Pr(T_{\mathrm{ij}}=1|L_{i})$

and where L_i_ denotes the latent classes of interest, such that $\sum_{Li=1}^{4} Pr(L_{i})$ = 1. *L_i_* = 1 denotes EPTB-positive, non-specific measurand-positive, *L_i_* = 2 denotes EPTB-positive, non-specific measurand-negative, *L_i_* = 3 denotes EPTB-negative, non-specific measurand-positive, and *L_i_* = 4 denotes EPTB-negative, non-specific measurand-negative.

The probability of a positive test result conditional on each latent class is expressed as follows:

$\Pr\left( T_{\mathrm{ij}}=1 | L_{i}=l \right)=\Phi(a_{jl}+b_{jl}r_{i})$, where $\Phi$ denotes the cumulative normal distribution function, a_jl_ and b_l_ are unknown parameters to be estimated and r_i_ ~ N(0, 1) is a subject-level random effect.

The following constraints were placed on the a_jl_ and b_l_ parameters to reflect that the accuracy of each test was only determined by its own measurand:

a_j1_ = a_j2_ and a_j3_ = a_j4_ for culture, Xpert and smear

a_j1_ = a_j3_ and a_j2_ = a_j4_ for the non-specific tests, cytopathology/histopathology and ADA

b_j1_ = b_j2_ for culture, Xpert and smear for all forms of extrapulmonary TB

b_j1_ = b_j2_ for cytopathology/histopathology for TB lymphadenitis

b_jl_ = 0 for ADA when l=1 or l=2

b_jl_ = 0 for all tests when l=3 or l=4

Prior distributions:

The following non-informative prior distributions were used:

L_i_ ~ Dirichlet((1,1,1,1))

a_jl_ ~ Normal(0,1)

b_jl_ ~ Uniform(0,5)

To avoid label switching, we truncated the prior distributions over the a_4l_ values such that they covered values greater than 0 when l=1=3 and covered values less than 0 when l=2=4.

*MCMC sampling details*

Three MCMC chains were run. After an adaptation phase of 2000 iterations, followed by a burn-in phase of 5000 iterations, we retained 15,000 iterations in each chain. For all parameters of interest, we examined the trace plots, the Gelman-Rubin R-hat statistic and the posterior density plots to determine if randomly selected initial values led to the same solution and convergence was achieved. Once convergence was achieved, we extracted the posterior median and equal-tailed 95% credible intervals for the parameters of interest.

*Model fitting and checking*

To examine how well the model agrees with the data used to fit it, we compared the different observed and expected frequencies of different patterns of test results. To verify if our approach to modeling conditional dependence was appropriate, we plotted the observed and expected pairwise correlation residuals (1). We also considered the probability of receiving treatment for extra-pulmonary TB versus the probability of extra-pulmonary TB, as estimated by the model.

*Identifiability*

To determine if a model was identifiable, we first checked if the number of unknown parameters was less than the available number of degrees of freedom. All models were fit with data on four observed dichotomous tests. Therefore, the number of degrees of freedom available in each case was 2^4^ – 1 = 15. For all models, the number of unknown parameters to be estimated was 12 (3 latent class probabilities, 8 a_jl_ parameters and 1 b_jl_ parameter). Therefore, the necessary condition for identifiability that the number of degrees of freedom exceed the number parameters to be estimated was satisfied.

We additionally checked for a sufficient condition for local identifiability by examining the rank of the Jacobian of the transformation from the joint probabilities of the test results to the parameters being estimated (sensitivity, specificity, prevalence parameters) (2). For the TB meningitis and TB pleuritis models we found that the criterion of local identifiability was not met as the rank was 10 (less than 12, the number of unknown parameters).

To detect possible problems with convergence and non-identifiability we examined the results of using non-informative prior distributions for all parameters. We encountered label-switching problems with some chains reaching solutions corresponding to (prevalence, sensitivity, specificity), while other reached (1-prevalence, 1-specificity, 1-sensitivity). We also noticed that the parameters of interest (prevalence of extrapulmonary TB or accuracy parameters with respect to extrapulmonary TB) appeared to converge in fewer iterations than estimates of the prevalence of the non-specific measurand and accuracy with respect to this measurand, particularly for the non-specific test. This could be because in all our models three tests measured the target condition whereas only one test measured the non-specific measurand. To fix the problem of label switching, we used truncated prior distributions over the sensitivity and specificity of the non-specific test with respect to its measurand. We also provided randomly selected starting values closer to the desired solutions. The Rhat statistics were close to 1 for all parameters once the label-switching problem was removed, suggesting good convergence. A visual examination of the posterior density plots (Supplementary Figure S2 A-C) shows that most parameters had posterior density plots with a single mode. The specificity of the non-specific test with respect to its measurand was the one parameter that seemed to have multiple values with the same posterior density (Supplementary Figure S2).

*rjags code for model likelihood and priors – TB lymphadenitis example*

model

# variables

#r: random effect

#p: matrix of probabilities for patient i and test j

#se: sensitivities

#d: true disease status

#sp: specificities

#a: intercept

#b: coefficient

# indices 1, …, 4 indicate culture, Xpert, smear microscopy, and ADA or cytopathology/histopathology

{

##--------------------- LIKELIHOOD -------------------------------------------------##

for (i in 1:N) {

for (j in 1:4) {

y[i, j] ~ dbin(p[LC[i],i, j], 1)

pp[i, j] <- pow( p[1,i,j], equals(LC[i],1) )*pow( p[2,i,j], equals(LC[i],2) )*pow( p[3,i,j], equals(LC[i],3) )* pow( p[4,i,j], equals(LC[i],4) )

}

LC[i] ~ dcat(pLC[1:L])

r[i] ~ dnorm(0,1)

# LATENT CLASS 1 : Target condition positive (EPTB+) and Measurand positive

p[1, i, 1] <- phi(a[1, 1] + b.RE[1] * r[i])

p[1, i, 2] <- phi(a[1, 2] + b.RE[2] * r[i])

p[1, i, 3] <- phi(a[1, 3] + b.RE[3] * r[i])

p[1, i, 4] <- phi(a[1, 4] + b.RE[4] * r[i])

# LATENT CLASS 2 : Target condition positive (EPTB+) and Measurand negative

p[2, i, 1] <- phi(a[2, 1] + b.RE[1] * r[i])

p[2, i, 2] <- phi(a[2, 2] + b.RE[2] * r[i])

p[2, i, 3] <- phi(a[2, 3] + b.RE[3] * r[i])

p[2, i, 4] <- phi(a[2, 4] + b.RE[4] * r[i])

# LATENT CLASS 3 : Target condition negative (EPTB-) and Measurand positive

p[3, i, 1] <- phi(a[3, 1])

p[3, i, 2] <- phi(a[3, 2])

p[3, i, 3] <- phi(a[3, 3])

p[3, i, 4] <- phi(a[3, 4])

# LATENT CLASS 4 : Target condition negative (EPTB-) and Measurand negative

p[4, i, 1] <- phi(a[4, 1])

p[4, i, 2] <- phi(a[4, 2])

p[4, i, 3] <- phi(a[4, 3])

p[4, i, 4] <- phi(a[4, 4])

}

##--------------------- PRIORS -----------------------------------------------------##

for (j in 1:3) {

a[1,j] ~ dnorm(0,1)

a[2,j] <- a[1,j]

a[3,j] ~ dnorm(0,1)

a[4,j] <- a[3,j]

}

a[1,4] ~ dnorm(0,1) T(0, ) # non-specific test sensitivity truncated above 0

a[2,4] ~ dnorm(0,1) T(,0) # non-specific test specificity truncated above 0

a[3,4] <- a[1,4]

a[4,4] <- a[2,4]

b.RE[1] ~ dunif(0, 5)

b.RE[2] <- b.RE[1]

b.RE[3] <- b.RE[1]

b.RE[4] <- b.RE[1]

pLC[1:L] ~ ddirch(prior[1:L])

for (i in 1:L) {

prior[i]<-1

}

##--------------------- PARAMETERS OF INTEREST -------------------------------------##

# SENSITIVITY AND SPECIFICITY WITH RESPECT TO TARGET CONDITION (EPTB)

# culture

se_cult <- ( phi(a[1, 1]/sqrt(1 + b.RE[1] * b.RE[1]))*pLC[1] + phi(a[2, 1]/sqrt(1 + b.RE[1] * b.RE[1]))*pLC[2] )/(pLC[1]+pLC[2])

sp_cult <- ( phi(-a[3, 1])*pLC[3] + phi(-a[4, 1])*pLC[4] )/(pLC[3]+pLC[4])

## xpert

se_xpert <-( phi(a[1, 2]/sqrt(1 + b.RE[1] * b.RE[1]))*pLC[1] + phi(a[2, 2]/sqrt(1 + b.RE[1] * b.RE[1]))*pLC[2] )/(pLC[1]+pLC[2])

sp_xpert <- ( phi(-a[3, 2])*pLC[3] + phi(-a[4, 2])*pLC[4] )/(pLC[3]+pLC[4])

# # smear

se_smear <- ( phi(a[1, 3]/sqrt(1 + b.RE[1] * b.RE[1]))*pLC[1] + phi(a[2, 3]/sqrt(1 + b.RE[1] * b.RE[1]))*pLC[2] )/(pLC[1]+pLC[2])

sp_smear <- ( phi(-a[3, 3])*pLC[3] + phi(-a[4, 3])*pLC[4] )/(pLC[3]+pLC[4])

# # cytopathology/histopathology (CH)

se_CH <- ( phi(a[1, 4])*pLC[1] + phi(a[2, 4])*pLC[2] )/(pLC[1]+pLC[2])

sp_CH <- ( phi(-a[3, 4])*pLC[3] + phi(-a[4, 4])*pLC[4] )/(pLC[3]+pLC[4])

# SENSITIVITY AND SPECIFICITY WITH RESPECT TO NON-SPECIFIC MEASURAND

# # cytopathology/histopathology (CH)

se_CH_measurand <- ( phi(a[1, 4])*pLC[1] + phi(a[3, 4])*pLC[3] )/(pLC[1]+pLC[3])

sp_CH_measurand <- ( phi(-a[2, 4])*pLC[2] + phi(-a[4, 4])*pLC[4] )/(pLC[2]+pLC[4])

# PREVALENCE OF EPTB AND NON-SPECIFIC MEASURAND

prev.EPTB <- pLC[1] + pLC[2]

# # prevalence of non-specific marker

prev.nonsp <- pLC[1] + pLC[3]

}

**SUPPLEMENTAL RESULTS**

*Model fit*

Table 1 displays the different test pattern results observed in lymph node samples from individuals tested for TB lymphadenitis. The observed counts of each test result pattern, along with expected counts, are shown, with generally good concordance. Exceptionally, there were fewer individuals observed with all positive test results except smear than the model expected (85 observed versus 98 expected), but the remaining patterns showed little discrepancy.

Pairwise residual correlations between test results are displayed in Supplementary figure 1. Residual correlation between tests was generally low, reflecting the observed conditional dependence was not more than expected under the model.

**Supplemental figure S1:** Pairwise residual correlation plots for tests run on A) lymph node, B) CSF, and C) pleural fluid. Test pair 1: culture and Xpert; 2: culture and smear microscopy; 3: culture and cytopathology/histopathology or ADA; 4: Xpert and smear microscopy; 5: Xpert and cytopathology/histopathology or ADA; 6: smear microscopy and cytopathology/histopathology or ADA. ADA – adenosine deaminase; CSF – cerebral spinal fluid; TB – tuberculosis.


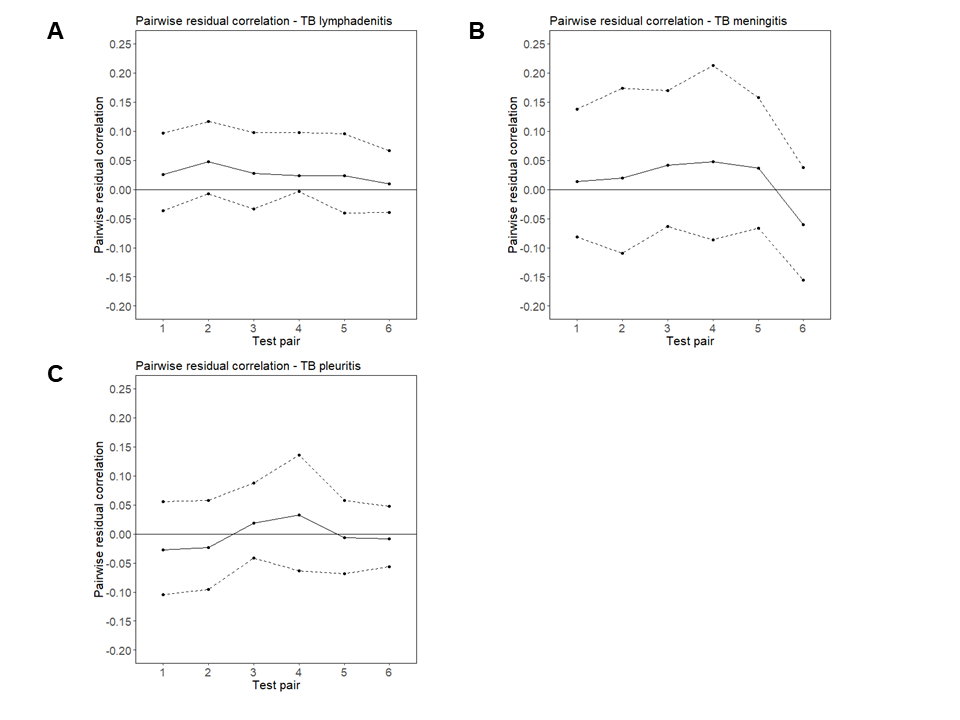


**Supplemental figure S2:** Density plots. For each of the three models (one per form of extrapulmonary TB), density plots were generated for each parameter of interest.

For test result parameters specificity, *C*, and sensitivity, *S*, the indices 1, …,4 indicate culture, Xpert, smear microscopy, and ADA or cytopathology/histopathology.

1. **TB lymphadenitis**


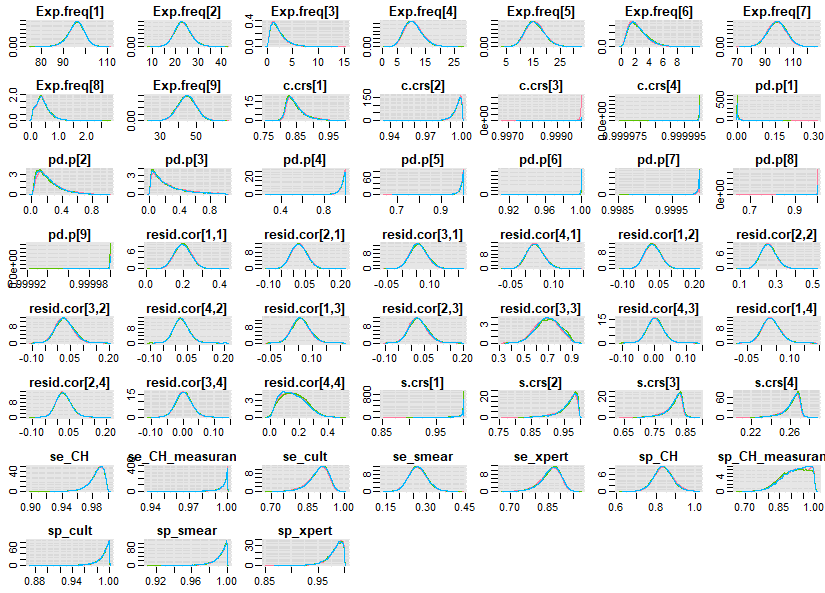


1. **TB meningitis**


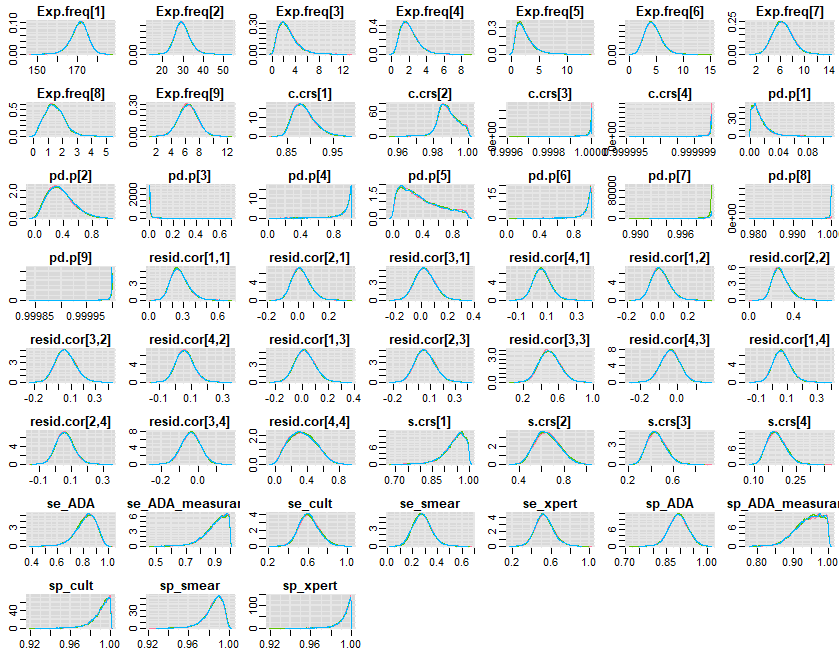


1. **TB pleuritis**


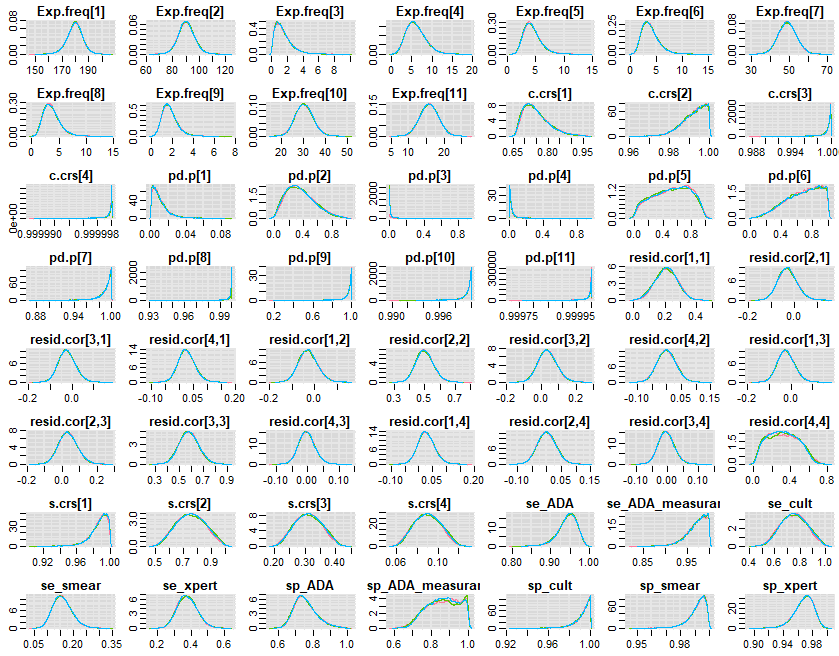


*Bayesian latent class analysis: estimated probabilities of the four latent classes*

**Supplementary Table S1**: Probabilities of the four latent classes, for each form of extrapulmonary TB as estimated by Bayesian latent class analysis.

|  | EPTB-positive, non-specific measurand-positive (“LC1”) | EPTB-positive, non-specific measurand-negative  (“LC2”) | EPTB-negative, non-specific measurand-positive (“LC3”) | EPTB-negative, non-specific measurand-negative  (“LC4”) |
| --- | --- | --- | --- | --- |
| TB lymphadenitis | | | | |
| Probability (95% CrI) | 0.60  (0.53, 0.67) | 0.0060  (0.0004, 0.023) | 0.030  (0.0010, 0.082) | 0.36  (0.29, 0.43) |
| TB meningitis | | | | |
| Probability  (95% CrI) | 0.14  (0.085, 0.22) | 0.013  (0.001, 0.048) | 0.043  (0.002, 0.13) | 0.79  (0.70, 0.87) |
| TB pleuritis | | | | |
| Probability  (95% CrI) | 0.31  (0.26, 0.47) | 0.010  (0.001, 0.036) | 0.073  (0.003, 0.21) | 0.55  (0.45, 0.69) |

The most common classes are EPTB-positive, non-specific measurand-positive and EPTB-negative, non-specific measurand-negative. The two classes where EPTB status and non-specific measurand are discordant were of far lower probability. This indicated that the four-class latent class model worked well to identify the target condition of interest in our study population, EPTB. CrI – credible interval; EPTB – extrapulmonary tuberculosis; TB – tuberculosis

*Two-class latent class model:*

**Supplementary Table S2**: Diagnostic accuracies of tests for each form of extrapulmonary TB by latent class analysis.

|  | Culture | Xpert | Smear microscopy | Cytopathology / Histopathology | ADA |
| --- | --- | --- | --- | --- | --- |
| TB lymphadenitis | | | | | |
| Sensitivity (95% CrI) | 90.4 (81.9, 95.4) | 86.9 (78.7, 92.4) | 27.4 (21.1, 34.3) | 98.9 (96.5, 99.8) | NA |
| Specificity (95% CrI) | 99.3 (96.3,100) | 98.4 (94.5,99.8) | 99.4 (97.0,100) | 84.8 (75.8, 95.2) | NA |
| TB meningitis | | | | | |
| Sensitivity (95% CrI) | 59.2 (41.7, 80.8) | 51.5 (35.0, 71.1) | 27.0 (14.0, 42.2) | NA | 87.8 (70.1, 97.1) |
| Specificity (95% CrI) | 99.2 (96.8, 100) | 99.5 (97.2, 100) | 98.6 (96.4, 99.7) | NA | 90.7 (83.8, 98.1) |
| TB pleuritis | | | | | |
| Sensitivity (95% CrI) | 69.3 (51.3, 91.8) | 35.0 (25.1, 47.8) | 14.2 (8.63, 22.3) | NA | 95.8 (90.4, 98.9) |
| Specificity (95% CrI) | 99.4 (97.3, 100) | 96.9 (93.8, 99.0) | 99.3 (97.5, 99.9) | NA | 79.0 (66.8, 98.6) |

**Supplementary Table S3**: Prevalence of each form of extrapulmonary TB as estimated by the two-class latent class model

|  | TB lymphadenitis | TB meningitis | TB pleuritis |
| --- | --- | --- | --- |
| Prevalence (95% CrI) | 60.4%  (54.0–67.0) | 16.2%  (9.65–25.0) | 38.0%  (27.6–51.5) |

**REFERENCES**

1. Qu Y, Tan M, Kutner MH. Random effects models in latent class analysis for evaluating accuracy of diagnostic tests. Biometrics. 1996;52(3):797-810.

2. Goodman LA. Exploratory latent structure analysis using both identiﬁable and unidentiﬁable models. Biometrika 1974; 61:215–231
